# The contribution of intrinsically disordered regions to protein function, cellular complexity, and human disease

**DOI:** 10.1042/BST20160172

**Published:** 2016-10-19

**Authors:** M. Madan Babu

**Affiliations:** MRC Laboratory of Molecular Biology, Francis Crick Avenue, Cambridge CB2 0QH, U.K.

**Keywords:** alternative splicing, biological networks, gene expression and regulation, intrinsically disordered proteins, protein turnover, RNA localization

## Abstract

In the 1960s, Christian Anfinsen postulated that the unique three-dimensional structure of a protein is determined by its amino acid sequence. This work laid the foundation for the sequence–structure–function paradigm, which states that the sequence of a protein determines its structure, and structure determines function. However, a class of polypeptide segments called intrinsically disordered regions does not conform to this postulate. In this review, I will first describe established and emerging ideas about how disordered regions contribute to protein function. I will then discuss molecular principles by which regulatory mechanisms, such as alternative splicing and asymmetric localization of transcripts that encode disordered regions, can increase the functional versatility of proteins. Finally, I will discuss how disordered regions contribute to human disease and the emergence of cellular complexity during organismal evolution.

## Introduction

**Figure 1.**
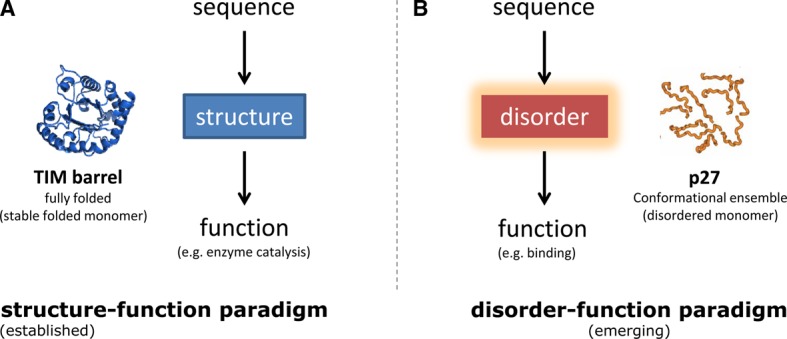
Sequence to function relationship. (**A**) Structure–function paradigm and (**B**) disorder–function paradigm. Reprinted with permission from ref. [7].

Understanding how proteins, which are polymers of amino acids, carry out different functions in a cell has been a problem of considerable interest. In a series of elegant publications, Christian Anfinsen and colleagues proposed that the sequence of a protein contains the information required to adopt a defined structure and, hence, function. This led to what is now called as Anfinsen's postulate or the thermodynamic hypothesis, which states that ‘the three-dimensional structure of the native protein in its normal physiological milieu is the one in which the Gibbs-free energy of the whole system is the lowest; that is, that the native conformation is determined by the totality of the interatomic interactions and hence by the amino acid sequence, in a given environment’ [1]. The biochemical studies of Anfinsen and colleagues, along with the unprecedented molecular insights obtained from crystallographic studies of proteins, such as hemoglobin and numerous enzymes, established the structure–function paradigm (Figure 1A). This concept postulates that function is achieved by the unique three-dimensional structure adopted by a protein, which in turn is determined by its amino acid sequence (see ref. [2] for a general historical overview).

While most proteins and polypeptide segments fold co-operatively into defined three-dimensional structures, numerous studies, primarily over the last couple of decades, have discovered that a large number of polypeptide segments do not fold into defined tertiary structure. Instead, they adopt an ensemble of different conformations and can still carry out their function in an unstructured/disordered state [3–6]. These studies are now establishing the disorder–function paradigm (Figure 1B), which states that certain polypeptide segments can be functional without achieving a defined tertiary structure [7–15]. Recent studies that have investigated genome sequences of many organisms have established that over 40% of any eukaryotic proteome contains such disordered regions [16–18]. More importantly, altered abundance and mutations in many proteins with disordered segments have been implicated in human diseases, such as neurodegeneration and cancer [19–28].

## Conformational states of intrinsically disordered regions

A major determinant of polypeptide segments folding co-operatively into a defined tertiary structure is the long-range hydrophobic interaction between amino acids in the linear sequence [29,30]. Intrinsically disordered regions (IDRs) are polypeptide segments that do not contain sufficient hydrophobic amino acids to mediate co-operative folding. Instead, they typically contain a higher proportion of polar or charged amino acids [31]. Thus, IDRs lack a unique three-dimensional structure either entirely or in parts in their native state. They generally sample a variety of conformations that are in dynamic equilibrium under physiological conditions [14,32–34].

**Figure 2.**
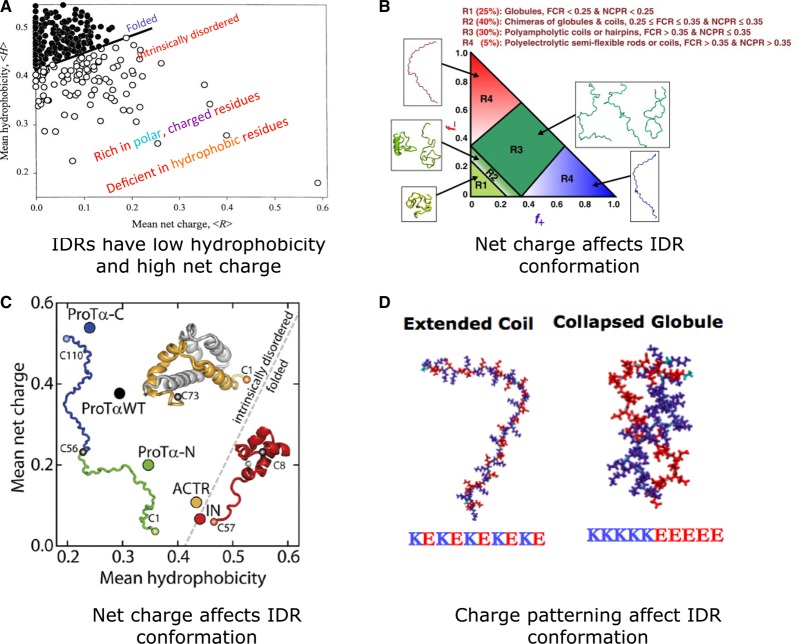
The relationship between sequence composition and conformations adopted by IDRs. (**A**) Plot of mean net charge versus mean hydrophobicity reveals the clear separation between structured proteins and IDPs. Reprinted with permission from ref. [31]. (**B**) Phase diagram showing the conformations of IDRs for different fractions of positive (*f*+) and negative charges (*f*−). Reprinted with permission from ref. [38]. FCR, fraction of charged residues; NCPR, Net charge per residue. (**C**) IDRs with sufficient hydrophobicity tend to fold upon binding (yellow, ACTR). Reprinted with permission from ref. [157]. ACTR, activator for thyroid hormones and retinoid receptors; ProTα-C, prothymosin α C-terminal segment; ProTα-WT, prothymosin α wild type; ProTα-N: prothymosin α N-terminal segment; IN, HIV integrase. (**D**) For the same net charge, the patterning can determine if the IDR adopts an extended coil or a collapsed globule conformation. Reprinted with permission from ref. [40].

This, however, does not mean that they are completely flexible and adopt all possible conformations. Computational analysis of sequences, single-molecule studies, and molecular dynamics simulations has revealed that the amino acid composition affects the IDR conformational states and can determine whether they adopt a totally extended conformation (segments with high net charge and low hydrophobicity) or a compact conformation (depending on the balance between hydrophobicity and net charge) [35–38]. This can further influence the functional elements (e.g. motifs or posttranslational modification sites) that are embedded within IDRs and can affect critical processes such as the cell cycle [39]. For the same number of charged residues, the charge patterning has also been shown to determine whether the polypeptide segment will be fully extended (e.g. alternating positively and negatively charged residues) or a collapsed globule (e.g. clearly separated stretches of positively and negatively charged residues), or somewhere in between (Figure 2) [38,40].

## Advantages and functions mediated by IDRs

**Figure 3.**
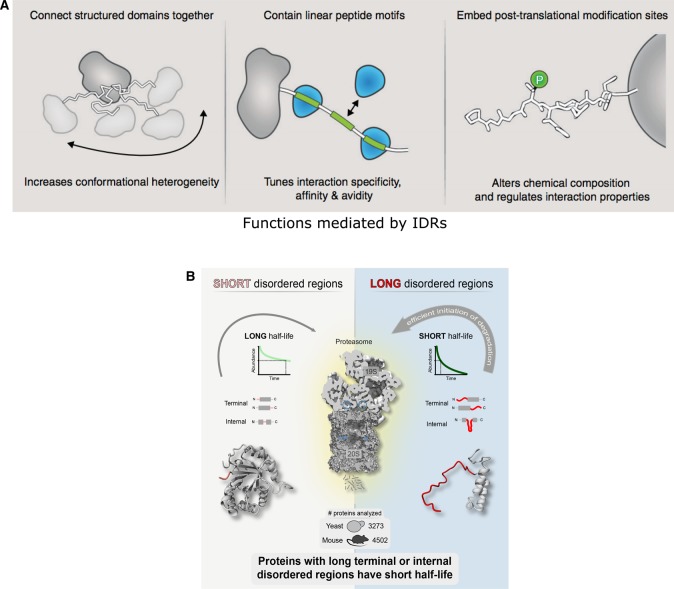
Advantages and functions mediated by IDRs. (**A**) IDRs can link structured domains, where their flexibility permits the protein to adopt multiple conformations; linear motifs within IDRs mediate protein interactions; posttranslational modification of residues within IDRs permits encoding and decoding of information [106]. (**B**) IDRs in protein sequences can increase the efficiency of degradation by the proteasome, thereby regulating protein half-life [53].

IDRs can provide many advantages to proteins (Figure 3). These include the following: (a) exposing short linear motifs that can mediate domain peptide interactions [41–44]. This permits interaction of the same protein with a large number of interaction partners in a functionally promiscuous manner or assembly of multiple proteins by serving as a scaffold (e.g. as seen in the AP2 adaptor protein during endocytosis [45,46]). (b) Facilitating the regulation of protein function via diverse posttranslational modification (PTM) of residues within the IDR [42,47,48]. Owing to their conformational flexibility, IDRs serve as excellent substrates to encode and decode information via posttranslational modifications (e.g. as seen in the tails of histone proteins or in the cytoplasmic tails of receptor tyrosine kinases and GPCRs [49–52]). (c) Regulating protein half-life by efficiently engaging proteins that have been targeted for degradation by the proteasome [53–59]. (d) Adopting different conformations when binding to different interaction partners [12,60–66]. These properties of IDRs make them well suited to perform signaling and regulatory functions. Indeed, genome-scale analyses of the functions of proteins with IDRs have revealed that they are enriched in signaling proteins and nucleic acid-binding proteins such as kinases, transcription factors and splicing factors [67–70].

## Folding upon binding of IDRs

**Figure 4.**
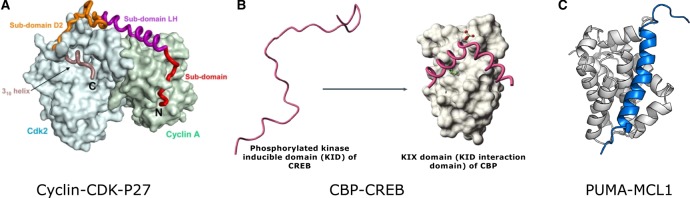
Coupled folding and binding of IDRs. (**A**) p27–cyclin–CDK complex. Reprinted with permission from ref. [158]. (**B**) CBP–CREB interaction regulated by phosphorylation. Reprinted with permission from ref. [32]. (**C**) PUMA–MCL1 interaction in apoptosis. Reprinted with permission from ref. [159].

An important aspect by which IDRs contribute to protein function is by adopting a defined conformation when binding a specific interaction partner [6,9,32,34,53,71–74]. Although a large fraction of the polypeptide adopts a defined structure upon complex formation, distinct segments can still remain disordered. This phenomenon has been referred to as fuzzy complex formation [75–78]. The folding and binding of IDRs facilitates interaction with their targets with relatively high specificity and low affinity [79,80]. This can permit highly specific associations to trigger signaling events while facilitating rapid disassociation when signaling is completed (e.g. p27 interaction with cyclin–CDK during the cell cycle; Figure 4A) [80]. The low-free energy of binding is due to the fine balance associated with the high entropic cost of folding and a comparable enthalpic gain of binding [60,72]. Thus, small perturbations either to entropy or to enthalpy of binding, such as via posttranslational modifications, can trigger association or disassociation from their interaction partners (e.g. CBP–CREB interaction; Figure 4B) [72]. While ‘weak but specific’ binding is often observed for IDRs, they also display very tight binding in several cases, which is often overlooked [81]. In terms of the kinetics of interactions, such proteins can have a wide spectrum of association and disassociation rates depending on the mode of interaction (e.g. conformational selection versus induced folding) [65,80,82–84]. For a given *K*_d_ value, the kinetic constants can vary widely [81]. Several different intrinsically disordered proteins (IDPs; proteins with IDRs) have exploited this property in order to facilitate robust cellular decision-making (e.g. as seen in the PUMA–MCL1 interaction involved in apoptosis; Figure 4C) [85].

## Formation of higher-order assemblies by IDRs

**Figure 5.**
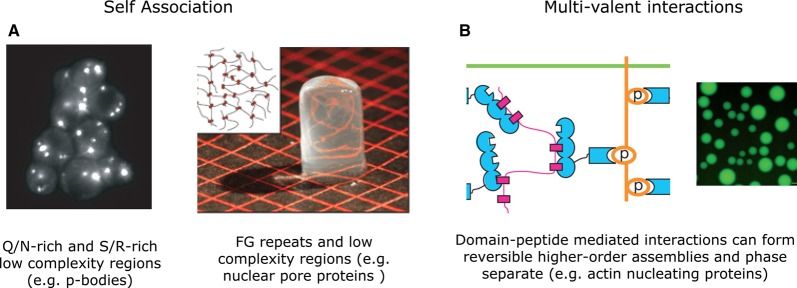
Formation of nonmembrane-bound organelles and higher-order assemblies by IDRs. (**A**) Self-association. Q/N-rich regions are important for P-bodies. Reprinted with permission from ref. [160]. FG-rich regions form hydrogel-like structures at the nuclear pore. Reprinted with permission from ref. [100]. (**B**) Multivalent interactions. Contacts between proteins containing repeating domains and peptide motifs can mediate phase transition that can be regulated via posttranslational modification. Reprinted with permission from ref. [92].

Recently, it has been demonstrated that many low-complexity regions and IDRs with repeating peptide motifs can form nonmembrane-bound organelles and higher-order assemblies, often in a highly reversible manner [86–98]. For instance, Q/N-rich regions are important for forming cellular assemblies, such as P-bodies, FG-rich regions are critical in forming the hydrogel-like structure of the nuclear pore, and repeats of multiple linear motifs can mediate phase separation and organize matter in cells, as seen in certain actin regulatory proteins (Figure 5) [92,99–102]. Thus, IDRs can mediate functions comparable to structured domains, such as (i) the formation of protein complexes and higher-order assemblies of variable stoichiometry of subunits [86], (ii) conformational transition (disorder-to-order and order-to-disorder) in response to specific environmental changes, context, or ligands [94], and (iii) allosteric communication [15,60,103–105]. Since most proteins contain structured and disordered regions in varying proportions, together with structured domains in the same polypeptide chain, IDRs can synergistically increase the functional versatility of proteins [12,15].

In this award lecture review, I will first describe emerging ideas on how alternative splicing of disordered regions can rewire protein interaction networks in a tissue-specific (TS) manner, thereby leading to increased complexity and diversity of interactomes of different tissues [106,107]. I will then describe our studies on how asymmetric mRNA localization and local translation of transcripts encoding IDRs can facilitate organization of higher-order assemblies in distinct parts of the cell and contribute to increased fidelity of signaling networks [108]. Finally, I will discuss how altered regulation and mutations within IDRs can cause many diseases [24,27,109].

## Splicing of disordered regions and functional versatility

Alternative splicing is a molecular mechanism that results in the formation of multiple transcripts from the same gene. In this manner, alternative splicing increases the potential number of distinct protein products that can be encoded by a single gene [110–119]. Many next-generation sequencing studies have established that over 90% of human genes are expected to undergo alternative splicing [111–113,120,121]. Interestingly, these studies also estimate that nearly 50% of the isoforms are likely to be expressed in a TS manner [113,120]. While high-throughput studies have established the extent of splicing at the transcript level, the roles played by the different variants at the protein level are not fully understood. In one of our studies, we investigated the characteristics of tissue-specific spliced exons and how they could have an impact on the function of the encoded protein. To this end, we systematically collected the complete transcriptome sequence of 10 human tissues and 5 human cell lines [120] and classified the exons into three groups: those that are (a) constitutively expressed, (b) alternatively included or excluded but expressed in multiple tissues, and (c) alternatively included/excluded but in a tissue-specific manner. We then investigated the structural properties of the encoded protein segment of these exons, analyzed their functional features (such as linear motifs and PTM sites) and evolutionary conservation, and studied various properties in terms of the protein interaction networks that they participate in within different tissues [107].

This analysis allowed us to make many observations. First, <5% of the TS exons map to complete protein domains, suggesting that protein segments encoded by TS exons are unlikely to adopt a defined tertiary structure. Further investigation revealed that such segments are enriched in disordered regions, which contain linear motifs and PTM sites that are evolutionarily conserved between human and mouse orthologs [107]. A detailed analysis of the proteins containing such segments revealed that they tend to have more interaction partners and mediate a higher number of TS interactions in the respective tissues where they are known to be alternatively spliced. Collectively, these observations suggested that disordered TS segments are unlikely to be passive linkers that connect structured domains, but have the potential to mediate new interactions via peptide motifs and PTM sites [107].

**Figure 6.**
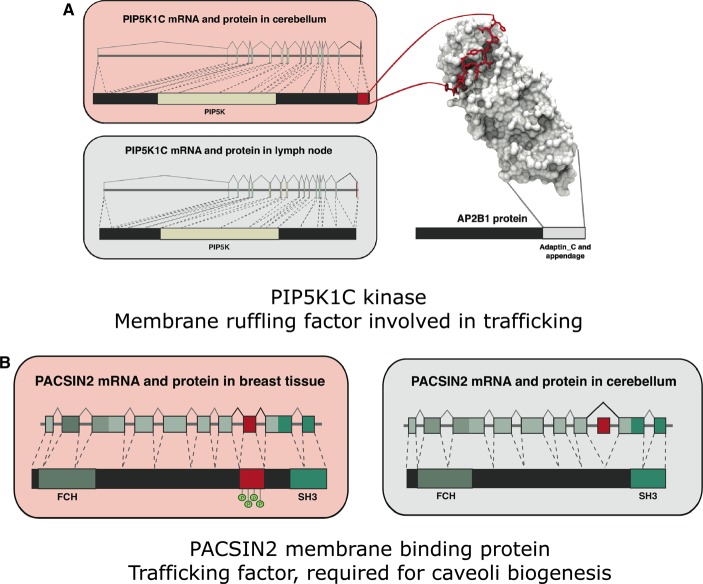
TS splicing can rewire protein interaction and signaling networks. TS splicing of IDRs can (**A**) affect interactions with other proteins by differential inclusion of linear peptide motifs and (**B**) influence whether a signaling enzyme can regulate a protein by differential inclusion of IDRs that contain posttranslational modification sites. Reprinted with permission from ref. [107].

**Figure 7.**
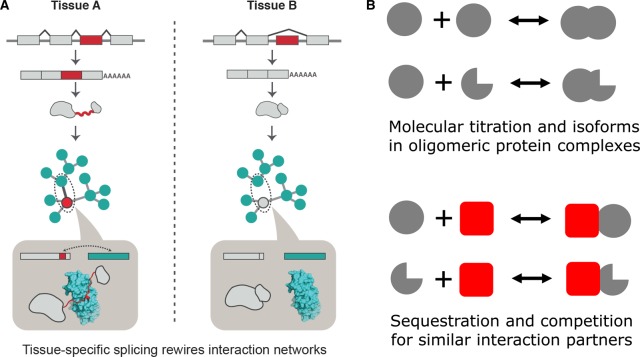
Impact of TS splicing of IDRs on protein networks and complexes. (**A**) Rewiring of protein interaction networks and signaling pathways by TS splicing. Reprinted with permission from ref. [107]. (**B**) Expression of multiple isoforms can affect response kinetics and influence cellular decision-making (ultra-sensitive behavior, dominant-negative response, and sequestration of interacting partners, leading to gain or loss of function).

To understand the molecular details of how TS exons can affect protein interactions, we systematically mapped the TS exons onto the known three-dimensional structures of proteins and protein complexes that were available in the Protein Data Bank [107]. This allowed us to describe the following general principles. TS splicing of disordered regions that contain (a) binding motifs can rewire protein interactions and thus contribute to the specificity of an interaction (Figure 6A) and (b) posttranslational modification sites can rewire signaling networks and make the protein a substrate for specific signaling proteins in a TS manner (Figure 6B). In this way, TS splicing of disordered segments can contribute to the rewiring of protein interactions and signaling networks in a TS manner and increase the diversity of protein networks in different tissues (Figure 7A) [107].

In addition to affecting protein interactions, such splicing events can also affect protein complexes. More specifically, expression of two different isoforms that can still interact (e.g. through the protein segment encoded by a constitutive exon) can lead to hybrid complexes. Depending on the region that is spliced, different isoforms can sequester and compete for the same interaction partner, which can lead to dominant-negative response, ultra-sensitive response, or transient gain- or loss-of-function effects — depending on which proteins are sequestered into nonfunctional complexes (Figure 7B) [107]. This is highlighted by the expression of an isoform of p53, which contains the DNA-binding domain but not the transactivation domain, a disordered region that is required to recruit the transcriptional machinery [107,122]. Expression of this isoform competes for the same regulatory elements in the genome in the promoter region of the p53 target genes, but ends up repressing gene expression of the targets since the transactivation domain is missing in this isoform. This mechanism has been exploited during development, by influencing pluripotency and differentiation of embryonic stem cells [122].

In this manner, TS splicing of disordered segments leads to the recruitment of the same biochemical activity (often carried out by structured domains encoded by constitutive exons) to different molecular contexts by mediating new protein interactions through the differentially spliced unstructured segment [107]. For example, even though the substrate protein may be expressed in a certain tissue, the TS inclusion or exclusion of a disordered substrate-docking motif in kinases can determine whether the kinase domain can phosphorylate a substrate protein or not. Similarly, even though the kinase may be expressed in the cell type, the TS inclusion/exclusion of a disordered modifiable residue within a substrate can determine whether it can be regulated by that particular kinase or not. In the case of transcription factors, splicing of the disordered transactivation domain in a TS manner or during specific times in development can convert a transcription factor from an activator to a repressor and hence, the same set of target genes can show very different transcriptional responses in different tissue types [107].

A number of related studies have all independently described similar observations [106,111,119,123–130]. Taken together, these studies reveal that alternative splicing of disordered segments can have important consequences [without affecting structured domain(s)] by rewiring signaling and regulatory networks in different cell types or during development. In this way, they increase the functional versatility of proteins by providing new contexts and expand the diversity of interaction networks in the different tissue types or at different time points during development. The plasticity associated with the divergence of alternative splicing between different organisms may have led to the emergence of novel phenotypes and increased complexity during organismal evolution [121,131,132].

## Localized translation of IDPs and cellular complexity

**Figure 8.**
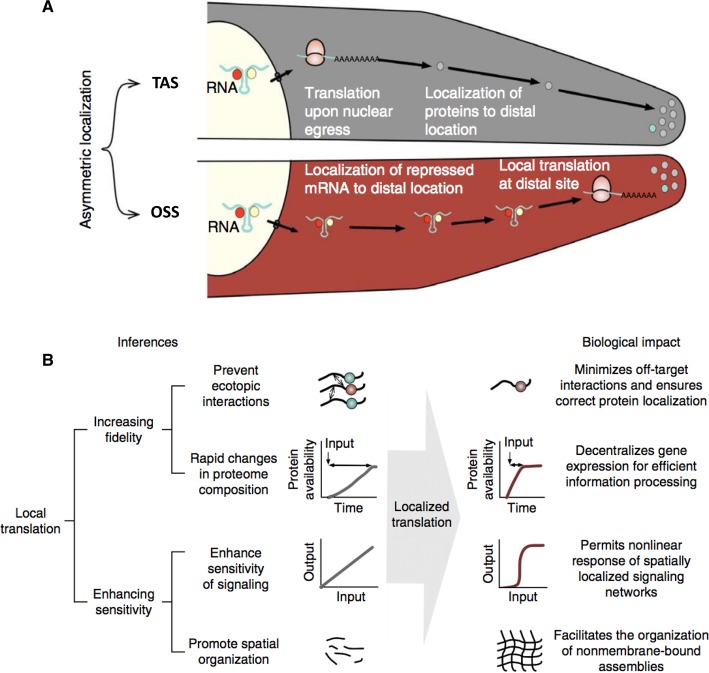
Asymmetric localization of proteins with IDRs. (**A**) Mechanisms to achieve asymmetric protein localization — transported after synthesis (TAS) or on-site synthesis (OSS) after asymmetric mRNA localization. (**B**) Advantages and implications of localized translation upon asymmetric mRNA localization. Reprinted with permission from ref. [108].

Asymmetric localization of proteins is a key to a wide variety of functions ranging from signal transduction in neurons and asymmetric cell division during development to maintaining cellular morphology [133–135]. The importance of asymmetric localization is apparent when localization goes awry, thereby leading to developmental defects and disease. For instance, in *Drosophila* embryos, mislocalization of *Oskar* mRNA to the anterior side produces embryos that have two abdomens with mirror-image symmetry [136]. Many studies over the last years have identified that there are two major mechanisms by which asymmetric localization of proteins can be achieved: The first mechanism involves protein transport after synthesis (translation), whereas the second one involves transport of the mRNA to specific locations in the cell followed by localized translation (Figure 8A) [134,137–142]. Both mechanisms can generate asymmetric localization of proteins and often operate in the same cell type [134].

Are there differences between proteins that are transported after synthesis (TAS) compared with those that undergo on-site synthesis (OSS) after mRNA transport? To investigate this question, we first compiled multiple large-scale datasets that experimentally identified asymmetrically localized transcripts and proteins in fibroblasts, mouse neuronal cells, and rat dorsal root ganglion cells from embryo and adult [140,141,143–146]. We then systematically integrated multiple large-scale datasets that described the structural properties of the proteins, mRNA, and protein expression levels, half-life, and functional features of proteins and transcripts to uncover the underlying trends [108].

This integrated large-scale analysis allowed us to make many observations, based on which we described general principles of how localized translation of transcripts that encode disordered proteins can (a) enhance signaling fidelity and sensitivity and (b) increase cellular complexity through precise spatial localization of proteins and formation of nonmembrane-bound assemblies (Figure 8B). A systematic comparison of structural properties revealed that TAS proteins are enriched in structured domains, whereas OSS proteins are enriched in disordered regions. Furthermore, proteins that are synthesized on-site are enriched in repeating linear motifs that have the potential to form higher-order assemblies. Such proteins also tend to be posttranslationally modified either within the motif or just around the motif, suggesting that OSS proteins might direct the flow of information and regulate the formation of reversible assemblies by using posttranslational modifications to switch protein interactions on/off. In addition, there was enrichment for OSS proteins encoding low-complexity regions; specifically, Q/N-rich regions and FG repeat-rich regions, both of which can undergo phase separation and form reversible, nonmembrane-bound assemblies. Taken together, these observations suggested that in contrast with transport after synthesis proteins, OSS proteins encode disordered regions, which contain multivalent, assembly-promoting segments that are surrounded by posttranslational modification sites (interaction/PTM switches; [41,47,147]). The trends were consistent across different cell types, organisms, and developmental stages, suggesting that these observations are likely to be applicable to different organisms [108].

Given the potentially promiscuous nature of such proteins, we then investigated how their availability is regulated. A systematic analysis of the protein abundance, protein half-life, transcript abundance, and transcript half-life revealed that OSS proteins and their transcripts are tightly regulated at almost every stage along the process of gene expression compared with the TAS group of proteins. An investigation of how the abundance and the PTM status of the two groups of asymmetrically localized proteins change over time after stimulating cells revealed that OSS proteins tend to increase their abundance more rapidly after stimulation compared with the TAS proteins. In summary, these findings suggested that proteins that are synthesized on-site are generally present in low abundance and are tightly temporally regulated. However, upon receiving a signal (e.g. stimulation with growth factors), they display a rapid increase in abundance and distinct phosphorylation dynamics [108].

There are many implications of the observations described here. Since many of the proteins that are synthesized on-site are likely to mediate promiscuous interactions and form higher-order assemblies, spatial localization of their transcripts to where they are required and their synthesis on demand by local translation can significantly restrict the likelihood of off-target interactions. Furthermore, since asymmetric mRNA localization decentralizes gene expression by decoupling transcription and translation, such a mechanism ensures that cells can rapidly respond to signals at the site where the signal is received and can process information within specific sub-cellular locations. In this way, localized translation after mRNA transport can sharpen the sensitivity of signaling networks and lead to nonlinear input–output responses for efficient information processing. It also ensures that while the overall copy number of the regulatory and signaling proteins may be low in a cell, at specific locations their local concentrations can be sufficiently high to help mediate their function (Figure 8B) [108].

On-site synthesis of proteins could further act as a general mechanism to ensure that nucleating proteins are available at the right place, in appropriate amounts, and only when required. In this manner, spatial control by localized translation may play a central role in signaling, by enhancing interaction fidelity and sensitivity, and by minimizing noisy, off-target interactions. Thus, together with other modes of regulation and temporal cues, such as signal integration via posttranslational modification, spatial control of proteins by localized translation can have a significant impact on cell signaling [108].

## IDPs and disease

**Figure 9.**
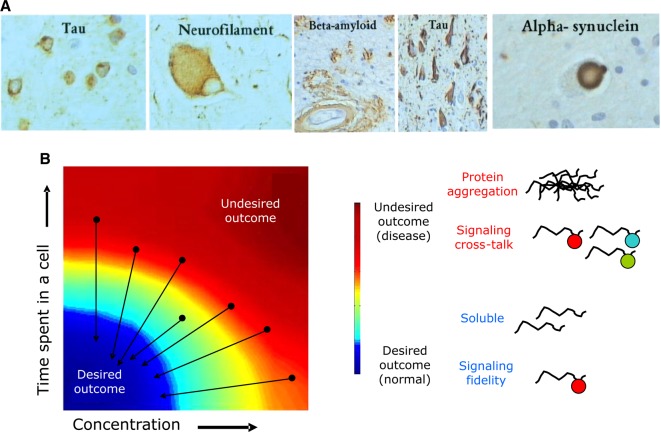
IDRs and disease. (**A**) IDRs are found in plaques and cellular deposits of patients with neurodegenerative disease. Reprinted with permission from ref. [161]. (**B**) Protein availability–outcome landscape. Tight regulation of proteins with IDRs (black arrow) ensures that they are present in the right amount and not longer than required. Adapted and reprinted with permission from refs [24,27,108].

While many studies have shown how proteins with IDRs can contribute to increased functional versatility and cellular complexity, research over the last several years has also revealed the importance of IDRs in many human diseases [19,24]. Mutations that lead to the alteration in the levels of proteins with IDRs can result in protein aggregation, leading to diseases such as neurodegeneration. Not surprisingly, it has been reported that aggregates of IDPs are found in very high concentrations in plaques and brain deposits of patients with neurodegenerative diseases (Figure 9A). Similarly, mutations within IDRs that increase the aggregation propensity, such as those seen in the amyloid β-peptide, α-synuclein, and huntingtin, have been directly linked to diseases such as Alzheimer's, Parkinson's, and Huntington's diseases, respectively [7,19,24,86,87,148–153].

It has been shown that IDRs are enriched in genes that participate in cell signaling and cancer-associated proteins, such as oncogenes or tumor suppressor genes [21]. Since IDRs typically contain motifs that can mediate low-affinity promiscuous interactions, altered abundance can form undesirable ectopic interactions and sequester other proteins into nonproductive complexes. In this manner, they can disturb the fine balance in many signaling and regulatory networks, leading to diseases such as cancer. Not surprisingly, gene fusions and missplicing of proteins with IDRs have also been associated with cancer [28,126,154]. Given our observations on the role of TS splicing and asymmetric localization of proteins with IDRs [107,108], it is likely that altered abundance of splicing factors and RNA-binding proteins that regulate the composition and the localization of mRNA may alter the protein sequence, localization, and availability of IDPs. This may result in off-target and potentially ectopic signaling events and might explain the molecular basis for cell type-specific disease phenotypes.

To address how the beneficial and potentially detrimental roles of proteins with IDRs are balanced in the cell, we investigated the availability of such proteins in a cell, both in terms of the time spent in the cell as well as in the steady-state amounts of IDPs and their transcripts in many organisms, ranging from yeast to human (Figure 9B). We and others have observed that proteins with IDRs are more tightly regulated than those with structured domains at multiple stages of gene expression, ranging from transcript synthesis to protein degradation [27,109,155]. In this manner, IDPs are tightly regulated to be present in the right abundance and for the appropriate amount of time in a cell. As long as this happens, the desirable outcome, such as interaction fidelity and solubility, is achieved. However, if their half-life or protein abundance is significantly altered, that may lead to undesirable outcomes such as protein aggregation or signaling cross-talk due to nonfunctional promiscuous interactions [24,27,109]. We suggested that, within a cell, a co-ordinated tight regulation of IDPs at several stages of transcription and translation ensures that they are present for short amounts of time and in low quantities [27,109]. This strategy minimizes the harmful effects of IDPs and at the same time permits their vital contribution to the functioning of the cell. An important implication of this observation is that, in addition to mutations that affect the IDPs and cause disease, mutations affecting genes that regulate IDPs availability can be an important class of disease genes that should be closely investigated in genome-wide association studies of human diseases [24,27].

## Conclusion

**Figure 10.**
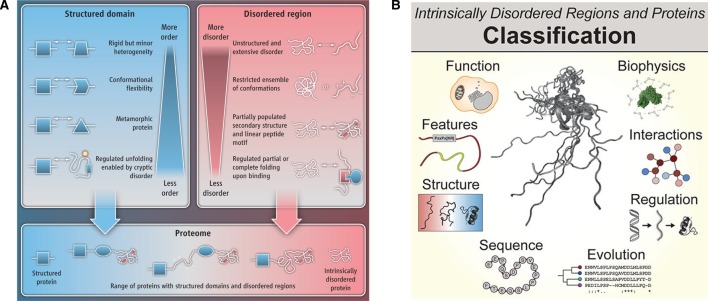
IDRs are fundamental units of protein function, regulation and evolution. (**A**) Synergy between structured domains and IDRs increases the functional versatility of proteins. Reprinted with permission from ref. [12]. (**B**) Classification of IDRs and IDPs. Reprinted with permission from ref. [7].

We have come a long way in our understanding of how proteins carry out their function in cells. In addition to the structured domains, which ensure precise positioning of side chains of specific amino acids in spatial proximity to carry out their function, IDRs, which adopt multiple conformational states, are emerging to be fundamental units of protein function and regulation. IDRs are not just passive linkers that connect different structured domains, but actively provide new contexts to structured domains and, hence, enhance the functional space associated with proteins (Figure 10A). IDRs are not all the same, but they can be classified into different groups based on various properties (Figure 10B) [7]. Since the number of functional residues in IDRs is small and clustered in the linear sequence (e.g. short linear motifs that mediate protein interactions), they can be gained and lost rapidly during evolution [41,42,156]. Thus, IDRs, in otherwise less evolvable proteins (e.g. developmentally important proteins), facilitate the exploration of new functional landscapes by changing the context in which the biochemical function can be applied. Therefore, disordered regions need to be studied in the right biological context to understand how complex functions emerge in cellular systems.

In conclusion, it is an exciting time for researchers who are investigating proteins with IDRs. Given the emerging importance of IDRs and a newfound understanding of their biomedical relevance, many discoveries regarding their myriad roles are yet to be unraveled. IDRs are now to researchers what the first few protein structures were to biologists half a century ago. We have witnessed the knowledge and impact on human health of the structure–function paradigm in the last 50 years. If structured proteins are only half the story, it brings to our attention the enormous possibilities and the potential of disordered proteins that remains to be tapped for bettering human health and revolutionizing medicine.

## Abbreviations

AP2, adaptor protein complex 2; CBP, CREB-binding protein; CDK, cyclin dependent kinase; CREB, cAMP response element binding protein; FG repeat, phenylalanine-glycine repeat; GPCRs, G-protein coupled receptors; IDPs, intrinsically disordered proteins; IDRs, intrinsically disordered regions; MCL1, myeloid cell leukaemia 1; OSS, on-site synthesis; P-bodies, processing bodies; PTM, post-translational modification; PUMA, p53 up-regulated modulator of apoptosis; TAS, transported after synthesis; TS, tissue-specific.

## Funding

None of this work would have been possible without the continued funding from the Medical Research Council [MC_U105185859] and support from EMBO, HFSP, BBSRC/ERASysBio+, the Royal Society, Trinity College, Darwin College, and the Lister Institute Research Prize.
